# Notes on Hydrogen Dioxide (H_2_O_2_)

**Published:** 1895-04

**Authors:** George S. Allan

**Affiliations:** New York City


### NOTES ON HYDROGEN DIOXIDE ($H_2O_2$).

BY GEORGE S. ALLAN, D.D.S., NEW YORK CITY.

HAVING of late made something of a study of hydrogen dioxide clinically, experimentally, and by means of expert testimony, and found that, much as has been written and said about it, there is still, in a measure, lacking in our profession a working knowledge of some of its properties and qualities. It seems proper to publish some notes taken, to correct errors as well as to invite discussion. Much care has been taken in obtaining information, and nothing has been accepted without careful examination.

There is no doubt whatever of the value of the agent or of the prominent place it will occupy in the dentists' "Materia Medica" in the future; therefore the more accurate our knowledge concerning it, the better it will be in all ways. There is a good excuse for many of the errors made by some of us, in the fact that the current literature of the day contains many misstatements of facts and theories, hence, of these we will take notice, prefacing our notes with a brief statement of what hydrogen dioxide really is and why it is so valuable.

Hydrogen dioxide, in its pure state, is a thick, syrupy fluid, but in that condition is a chemical curiosity only, as dangerous to handle as it is difficult to obtain. Dentists and physicians know it only in its solutions. It is known to chemists as an unstable compound, one ready to break apart. In its decomposition a molecule of it forms a molecule of water and an atom of oxygen. Water, as we all well know, is a stable compound, while oxygen, especially at the moment when it is freed from some former combination, is especially eager to enter into a new union. Its affinities are many and strong, and for few substances has it greater attraction than for some of the elements entering into the composition of disease-germs and decomposing animal or vegetable matter. These it at once attacks, breaking them up, uniting with some of their elements and forming new and harmless substances. Hydrogen dioxide is valuable only as it furnishes pure, fresh oxygen. The solvent ordinarily employed is water. To how great an extent water can hold hydrogen dioxide I do not know. The United States Pharmacopœia does not recommend aqueous solutions of a higher strength than three per cent.; the Pharmacopœia committee probably having found that aqueous solutions of a higher strength do not possess good keeping properties. Ethereal solutions, however, can be kept up to a strength of fifty per cent. A solution of this strength would, by the Pharmacopœia test, yield about one hundred and fifty times its volume of available oxygen. As to the strength of what might be called a saturated solution, and there is much difference of opinion as to the proper manner of estimating it, pure hydrogen dioxide will liberate some three hundred times its volume of oxygen when decomposed, not counting that held in the residue of water remaining. The United States Pharmacopœia standard preparation is a three-per-cent. solution, equivalent to a volumetric strength of ten volumes of available oxygen per one volume of solution; each one per cent. counting 3.33 of volume. It is an easy matter to test the strength of any aqueous solution by the method given in the last edition of the United States Pharmacopœia. The decinormal solution of permanganate of potassium there recommended can be obtained from Eimer & Amend, New York City, and must be used when freshly prepared. The three preparations, pyrozone, the hydrogen dioxide of the Oakland Chemical Company, and hydrozone, were thus tested by the Pharmacopœia process, and all of them were found to be of full strength. The first two corresponding to a ten-volume test and a point or two more. Marchand's hydrozone did not come up to their published statement as to strength, but the test was hardly a fair one, as the portion tested was what was left in a bottle that had been open for some time; as it was, the test indicated fully a twenty-two-volume solution. It should have indicated about a twenty-seven-volume solution. Hydrozone is about the strongest aqueous solution in the market, but not the most permanent.

So much has been written concerning the value of the various aqueous solutions that it does not seem wise to cover the ground again. As a means of discovering the presence of pus and of destroying it utterly when found, it does not seem to have an equal. The action of $H_2O_2$ solutions in the presence of pus and decomposing organic matter is about as follows:

"Pus is an extremely complex body, and but little is known as to its constitution. Schwilgue has made an approximate analysis of its constituents, and finds it to contain albumen and water, a peculiar extractive substance, and small amounts of soda, phosphate of calcium, and other salts. It is easily coagulable by heat, acids, alcohol, and other liquids.

"In adding a little pyrozone three-per-cent. solution to pus, vigorous effervescence takes place, a mass of white foam resulting, which is easily disintegrated by water; carbon dioxide and sulphur dioxide, or sulphurous acid, are at the same time liberated.

"The exact chemical reactions taking place are not easy to determine, but probably something as follows occurs: The albumen is instantly attacked by the nascent oxygen liberated from the hydrogen peroxide, on contact with the organic substance, and an action probably occurs similar to that taking place when albumen is treated with oxidizing agents or alkalies. Albumen contains CNHOS in indefinite proportions. Part of the carbon combines with the oxygen to form $CO_2$, and the sulphur likewise unites to form $SO_2$, the evolution of these gases causing the effervescence previously mentioned. The remainder of the carbon, nitrogen, hydrogen, etc., in the albumen, is converted into simpler bodies which are harmless and soluble. These would be principally bodies such as glutamic acid, leucine, tyrosine, aspartic acid, and ammonia. Were the action continued long enough, the ultimate products would be simply $SO_2$, $CO_2$, $N.H_3$, and $H_2O$; but it is unnecessary to proceed so far, the bodies formed being easily removed from a cavity by water.

"Expressed in the form of an equation, the reaction might be written,—

$$n\text{Pus} + nH_2O_2 = x\text{Albumen} + xSO_2 + xCO_2 + xH_2O \text{ and}$$

$$\text{Albumen } (C_{53}H_7N_{15}O_{21}S_1?)n + nH_2O_2 =$$

$$xSO_2 + xCO_2 + xNH_3 + \underset{\text{Glutamic acid.}}{xC_3H_5NH_2{<}^{COOH}_{COOH}} +$$

$$\underset{\text{Leucine.}}{xC_5H_{10}{<}^{NH_2}_{COOH}} + \underset{\text{Aspartic acid.}}{xC_2H_3(OH){<}^{COOH}_{CONH_2}} +$$

$$\underset{\text{Tyrosine.}}{xC_6H_4{<}^{OH}_{C_2H_3{<}^{NH_2}_{COOH}}} + xH_2O."$$

The evolution of gas that takes place in the above description of the chemical action is a most valuable help to the complete chemical disintegration of effete organic matter. By its means the said matter is broken up, its parts separated, and so more quickly and completely brought under the influence of the newly-born oxygen. As a mouth-wash, this means that the germicidal power of $H_2O_2$ is very great and that it will reach the interiors of masses of dead organic matter when other germicidal preparations, acting on the surface only and oftentimes actually forming on them a protecting coating, would fail to act, or at best act only imperfectly.

To the dentist the ethereal preparations of $H_2O_2$, as pyrozone five-per-cent. solution and pyrozone twenty-five-per-cent. solution, are most useful. Ether, as a solvent, possesses valuable properties. The solution is a stable one, and bottles containing it can be kept and handled as freely as if they contained ether only; either of the two preparations, the five-per-cent. or the twenty-five-per-cent. solution, can be evaporated seventy-five per cent. of their volume before the $H_2O_2$ is liberated. I have had numbers of bottles in more or less constant use during the last two months, and have had no trouble with them whatever. In no case, when drawing the stopper, have indications of undue pressure been observed.

The solvent properties of ether greatly aid in preparing the way for the action of the free oxygen. It readily dissolves the fatty matter contained in the carious portions of dentine; it penetrates the dental tubuli rapidly and effectually, carrying with it the $H_2O_2$, which completes the process of germ-destruction and bleaching. The evolution of gas in the tubuli forces the contents out and leaves them in a clean, healthy condition. To obtain the best effects, it is wise to use the five-per-cent. solution first, and instead of evaporating it, swab it out, using it somewhat liberally; it may be absorbed with bibulous paper, in this way much of the bulk of the dead matter may be removed before the final bleaching or germ-destruction is completed by the pyrozone twenty-five-per-cent. solution. The action of this last should be hastened and increased by means of the hot-air blast.

The ethereal preparations will tell many a story of defective adaptation of filling-material to the walls of cavities. Many times, when patching old fillings, the eye fails to detect trouble until the forming of the gas around the defective margins points it out; especially has this been found to be the case in old amalgam fillings. It is both safe and wise to make this test in all doubtful cases.

One of the commonest mistakes made in referring to the action of $H_2O_2$ is found in the constant reference to ozone as being either formed or liberated by $H_2O_2$ in the presence of decomposing organic matter. It is safe to say that this is never the case. Ozone is an allotropic condition of oxygen. Three volumes of oxygen form only two of ozone. The oxygen molecule is composed of two atoms of oxygen, the ozone molecule contains three. Oxygen is soluble in water, ozone is almost insoluble, and should it be found in water, it would be almost at once liberated from solution. The literature on this subject, especially that in the circular or other advertising forms, including many journal articles, contains many curious examples of this error. The terms "hydrozone" and "pyrozone" convey a false impression, and ought never to have been adopted, though they are but distinctive trade names. On the first page of the circular issued by Marchand will be found the statement, in large type, "Ozone is the healing agent of this remedy," referring to hydrozone; and further on, "Ozone being set free from the decomposition of the hydrogen coming in contact with the infected surface."

In a monograph published by the Oakland Chemical Company will be found the sentence, "In this connection it has been found that the peroxide (dioxide) of hydrogen, which is the subject of this brochure, is the readiest and most readily available and reliable source of free ozone or active oxygen yet discovered." Such erroneous statements as the above are hardly excusable.

The name Hydrozone is entirely a misnomer, indicating that the preparation contains ozone dissolved in water. Pyrozone, on the other hand, the makers inform me, is derived from the Greek word for fire, and is so named from a property possessed by anhydrous $H_2O_2$ of igniting a silk chenille ball when placed in contact with the pure substance procured from ethereal solutions. This term is applied not to the aqueous or ethereal solutions, but to the pure $H_2O_2$ itself, from which they are made.

An article going the rounds of the medical press contains some strange statements and deserves notice. It is headed, "The real value of medicinal preparations of hydrogen dioxide." Bearing in mind that the last edition of the United States Pharmacopœia states that the aqueous hydrogenii dioxide shall yield about ten volumes of available oxygen, and recognizes that as the official standard so far as volumetric strength is concerned, Dr. Endemann's "my opinion" that "a standard solution of medicinal $H_2O_2$ must contain at least fifteen volumes of available oxygen" seems rather out of place. Would the doctor have the reader place his authority as to what is the proper strength in advance of the United States Pharmacopœia? Again, referring to a certain brand of $H_2O_2$ solution, the doctor says it contains salicylic acid. The manufacturers of that brand deny that any of this acid can be found in their preparations, and the iron and ether test,—the usual one,—carefully made, entirely disproves the assertion of Dr. Endemann. Even trade criticisms ought to be fairly reliable.

Dr. Endemann, in a later article in the *Times and Register* on the keeping properties of solutions of $H_2O_2$, written apparently for the benefit of a particular maker's brand, shows conclusively that pyrozone three-per-cent. solution is the most stable brand of $H_2O_2$ on the market. The loss of available oxygen, according to his own table, in a sample after being kept three months, is less than two volumes per one hundred volumes, whilst the brand of the maker in whose interest the article appears to have been written, showed a loss of ten volumes per one hundred volumes. The freedom of the pyrozone solution from excess of acid, poisonous barium salts, and other impurities, was shown to be greater than any other brand on the market. Further, brands possessing a greater strength than that ordered by the United States Pharmacopœia are shown not to possess good keeping qualities, the pharmacopœial strength of three per cent., combined with freedom from excess of acid, being the requisites necessary to insure a stable permanent solution.

Pyrozone twenty-five-per-cent. ethereal solution can be used instead of sodium peroxide, and for the same purposes. It has the advantage of being always ready for use. The preparation of sodium peroxide requiring much care and attention on the part of the dentist, in a measure limits its value and advantages. At this writing, however, it is not possible to say which compound is best for dental purposes; the probabilities are that each compound has its special virtues.

Reading the article on electrozone in the February issue of the INTERNATIONAL DENTAL JOURNAL, I was easily satisfied that it was both weak and inaccurate, and did not fairly state either what it was or its mode of action. I therefore obtained a letter from Dr. Cyrus Edson, and also an opinion from an expert chemist. As they fairly present the subject, they are here appended.

"DR. GEORGE S. ALLAN, No. 51 West Thirty-seventh Street, New York City:

"DEAR DOCTOR,—In reference to electrozone: electrozone is a solution of hypochlorites, mainly, however, hypochlorite of sodium. It is produced by the action of electricity on sea-water. The current used is the Edison variety, low voltage, but high electromotive force, which is caused to act through electrodes of platinum and carbon. The result of this action is to decompose the salts contained in the sea-water, mainly sodium chloride, which is converted into sodium hypochlorite. This hypochlorite is a very unstable one, which is very readily decomposed by contact with organic matter. The result of the decomposition is the production at the point of contact of free oxygen and chlorine. These agents act on vegetable life instantly, destroying it. In a sense, electrozone is similar to Labarraque's solution, except that it, of course, contains no caustic soda. It is not poisonous in the ordinary sense of the term, and I believe is one of the most effective of disinfectants. Of course, a solution of hypochlorite produced in the way I have described may vary very considerably in strength, according to the percentage of chloride of sodium contained in the electrolyte, and the length of time and strength of the current applied. A solution of full strength should contain but a very small proportion of free chloride of sodium. Sea water containing four per cent. of chloride of sodium should have been so acted on by the current as to convert nearly all of the chloride of sodium into hypochlorite. Such a solution would be, in my opinion, a better disinfectant than a five-per-cent. carbolic acid solution. The great advantage, however, of the hypochlorite solution made by the action of electricity is its extreme cheapness, in which point no other disinfectant that I know of is comparable with it. Yours faithfully,

"Cyrus Edson."

EXPERT OPINION OF ELECTROZONE.

In electrolyzing pure water slightly acidulated, minute amounts of ozone and possibly also $H_2O_2$ are formed. The ozone, being insoluble in water, is at once liberated, and cannot exist as such in any aqueous solution. These bodies are not, however, formed when solutions of metallic salts, particularly halogen salts, undergo electrolysis, local action, and secondary decomposition, apart from the action of the liberated ions sufficing to decompose them if formed. The electrolytic action taking place when a solution of NaCl is decomposed may be thus expressed,—

$$NACl = NA + Cl$$

$$NA + H_2O = NAOH + H$$

$$2NAOH + Cl_2 = NAOCl + NACl + H_2O.$$

The ultimate products being hypochlorite of sodium with a little chloride and usually small amounts of free chlorine. Hydrogen is liberated at the negative pole. Carbon electrodes must be used to prevent the chlorine set free from attacking them. This in itself would prevent any formation of hydrogen peroxide.

ACTION OF ELECTROZONE DISINFECTANT.

Mr. Woolf's explanation (page 69) is ingenious, the only objection to it being the fact that it is altogether contrary to all chemical principles. The action of hypochlorites as disinfecting agents is not due to decomposition by organic matter, but to liberation of chlorine by acids, usually $CO_2$. In this way hypochlorous acid is set free, which is at once oxidized by the oxygen of the air to form chloric acid, chlorine being set free at the same time. Thus, $4HClO + 3O = 2HClO_3 + Cl_2 + H_2O$. Neither free chlorine nor hypochlorites will abstract hydrogen from water, as stated, nor will Cl necessarily liberate oxygen from organic matter. The "three atoms of nascent oxygen" exist, therefore, solely in the author's fertile imagination. Supposing they were formed, we are not aware that ozone consisted of "three atoms of nascent oxygen." Indeed, ozone is the exact opposite of nascent oxygen in composition, being a condensed form of that element, while nascent oxygen is oxygen in its simplest or atomic form.

ACTION ON GERMS.

"All germs subjected to the action of the electrical disinfectant are contracted, on account of the hydrogen being deprived of one element." This may be so, but we are not aware of it. Neither are we aware that hydrogen is a compound body and can be "deprived of one element." What element? The contraction of the germ, on the application of a saline solution, is easily understood by any one having the faintest knowledge of physiological botany. The plasmolytic action of certain salts on the vegetable cell is one of the easiest experiments to make, and one of the easiest to explain.

We do not see that the alteration in the shape of a dead germ adds anything to the value of a disinfectant, and whether the germ be round or long, so long as it is dead, it can do no harm.

Mr. Woolf advances a new test for the presence of bacteria, and a new proof that toothache is caused by bacteria. This, he says, can be proved because electrozone will cure it!

ACTION ON VEGETABLE COLORS.

According to the inventor, these are absolutely destroyed. Any tyro can prove the fallacy of this statement. The fact that any oxidizing agent will decolorize any such solution is well known, as also the fact that the color can be restored by the action of reducing agents. The organic matter is still there, it has been only slightly altered. Similarly other organic matter is merely oxidized to some other form, usually harmless, by disinfectants. To say that it is entirely destroyed is inaccurate.

"The scientists to-day claim that all germs have their origin in decayed vegetation." This is a revival of the long-since exploded theory of biogenesis.

The chemistry of the whole paper is lamentably weak, and the statements loose and inaccurate, even where the author's meaning is clear. For instance, "coal-oil is not obtained from carbon, neither are aniline dyes obtained from coal-oil," but from coal-tar.

COAGULATION OF GERMS (page 71).

These so-called disinfectants ($HgCl_2$, $C_6H_5OH$, and $SO_2$), says the author, form a coagulated coating of albumen on the outside of the germs, which are rendered temporarily inert. On the disintegration of the coagulated coating the *germ comes to life again!* It is idle work trying to prove that $HgCl_2$ and $SO_2$ are not disinfectants, when it is well known that $HgCl_2$ is more powerful than even free chlorine.

HEAT ON ELECTROZONE.

The author shows his great deficiency of knowledge of chemistry when, in reply to a question, he says that he does not know what the effect of heat on the solution would be. This is one of the first things a student of chemistry would learn, that on heating a solution of a hypochlorite, decomposition takes place with formation of a chlorate and chloride, thus rendering the disinfectant useless. $H_2O_2$ in solution has the advantage here inasmuch as it can be heated without risk of decomposition.

